# Development and validation of a digital biomarker for peripheral artery disease

**DOI:** 10.1038/s41746-026-02655-w

**Published:** 2026-05-12

**Authors:** Mattheus Ramsis, Ava J. Fascetti, Mustafa H. Naguib, Shamim Nemati, Pam R. Taub, Christopher A. Longhurst, Elsie G. Ross, Edward J. Wang

**Affiliations:** 1 Division of Cardiology, Department of Medicine, University of California, San Diego, CA USA; 2 The Design Lab, University of California, San Diego, CA USA; 3 Department of Electrical and Computer Engineering, University of California, San Diego, CA USA; 4 School of Medicine, University of California, San Diego, CA USA; 5 Department of Biomedical Informatics, University of California, San Diego, CA USA; 6 Department of Medicine, University of California, San Diego, CA USA; 7 Division of Vascular Surgery, Department of Surgery, University of California, San Diego, CA USA

**Keywords:** Biomarkers, Cardiology, Diseases, Medical research

## Abstract

Peripheral artery disease (PAD) is a common manifestation of atherosclerotic cardiovascular disease (ASCVD) that is underdiagnosed in clinical practice. Photoplethysmography (PPG) serves as a widely available tool that captures peripheral vascular physiology, yet the quantitative links between PPG signal characteristics and the presence of PAD are underexplored. In analyzing 5,237 legs from *N* = 2362 unique patients, we find significant correlations with multiple PPG features and the ankle-brachial index (ABI), a commonly used non-invasive diagnostic test for PAD. Using these explainable features, we develop a machine learning model to detect PAD solely from PPG features (AUC = 0.83) and develop an enhanced model incorporating clinical information (AUC = 0.85). Additionally, our model is highly generalizable, performing similarly across demographics and comorbidities. These findings represent an initial step toward identifying an accessible, physiologically grounded digital biomarker associated with PAD, and lay the foundation for prospective studies to evaluate performance across clinical workflows and reference standards.

## Introduction

Peripheral artery disease (PAD) is a condition characterized by plaque accumulation in the arteries, particularly of the lower extremities, affecting an estimated 12 million Americans and 200 million adults worldwide, significantly increasing the risk of limb loss and major adverse cardiovascular events^1–3^. Despite its prevalence, PAD is an underdiagnosed condition, with most patients diagnosed at a more advanced stage of disease. This is especially apparent among high-risk populations where health disparities exist, leading to substantial medical and financial burdens for patients and the US healthcare system^2,4–6^. Despite its limitations, the gold standard and cornerstone of diagnosis of PAD includes the non-invasive ankle-brachial index (ABI), first described as a test for the disease in the 1950s–60s^7^. There has been a lack of innovation in diagnostic tests for PAD over the past 60 years. In addition, there continue to be major barriers to the use of the ABI for the detection of PAD in under-resourced populations, including the lack of time (with measurements taking 10–15 min), lack of staff, and reimbursement^6,8,9^. A survey of US primary care practices found that nearly 70% of providers reported never using ABI, with only 12–13% using it weekly or monthly^8,10^. Given the lack of widespread innovations to improve the detection of PAD and the increasing prevalence of the disease, especially in underserved communities, there exists a glaring unmet clinical need to develop technology to meet the demands of modern practice.

The ABI is the most widely used noninvasive test for the evaluation of PAD and forms the current basis of epidemiologic studies, clinical guidelines, and is used as the primary comparator in the vast majority of PAD treatment trials^7,11,12^. Abnormal ABI values have been consistently associated with increased risk of cardiovascular events, limb outcomes, and mortality, supporting its established prognostic significance^13–15^. Although ABI has recognized limitations in diagnostic sensitivity, particularly in patients with distal disease, diabetes, chronic kidney disease, or non-compressible arteries, it remains the cornerstone and initial reference standard for PAD diagnosis and risk stratification in routine clinical practice^7^. These limitations motivate interest in complementary physiologic markers that may augment existing assessment strategies rather than replace them.

Photoplethysmography (PPG) offers a potential non-invasive, widely accessible solution for PAD detection. PPG technology involves a light that shines into tissue, such as the fingertip, wrist, or toe, and quantifies the backscattered light that corresponds with changes in blood volume^16^. As PPG technology is readily available on all smartphones, noninvasive wearables, and mobile or in-clinic pulse oximeters, it is also foreseeable that a PPG-based solution can potentially be adopted as a widespread tool directly to patients to diagnose unrecognized disease. PPG is well-positioned to capture the multifactorial intravascular sequelae of PAD, including metabolic changes, endothelial dysfunction, and disturbances in vascular tone^17–19^. In fact, PPG technologies have already been utilized to detect chronic cardiovascular and metabolic conditions, including diabetes and atrial fibrillation^17,18^. However, this avenue has not been sufficiently explored in the digital detection of PAD. Previous work has explored the potential for deep learning to detect PAD from PPG signals; however, this work was limited to a small sample size, minutes-long PPG signals, and lacked transparency^20^.

In clinical practice, some vascular laboratories routinely obtain an ABI in conjunction with PPG measurements as part of the PAD assessment^21^. Given this, the toe PPG (T-PPG) waveform that is captured, combined with a machine learning (ML) algorithm, can potentially be leveraged to more easily identify PAD^22^.

This study was designed to use previously obtained T-PPG waveforms as part of routine care from two centers in the University of California San Diego (UCSD) Health System to: (1) explore the relationship between PPG signals and PAD to unpack which features of PPG correlate with an ABI-defined PAD diagnosis; (2) develop and validate a ML model to detect PAD from PPG signals; (3) determine how PAD detection performance changes across different demographics and comorbidities.

## Results

### Study cohort

**Fig. 1:**
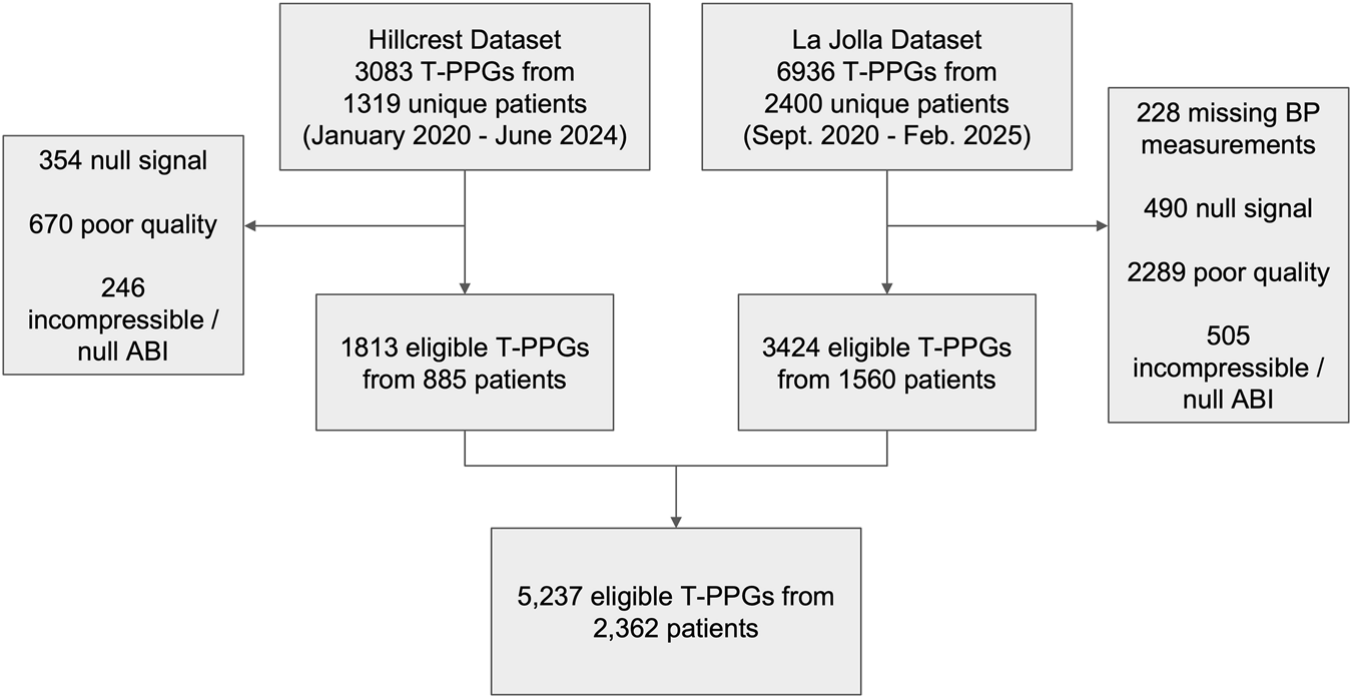
Multi-database study cohort of toe photoplethysmography (T-PPG) measurements. Signals were removed due to missing blood pressure (BP) measurements, incompressible ankle-brachial index (ABI) values, or due to poor four-second T-PPG recordings. Note that 83 patients were shared across each database after exclusion. Excluded signals did not bias the dataset toward certain patient groups or peripheral artery disease (PAD) severity levels, see Supplemental Fig. 1 for details on excluded signals.

We curated a dataset comprising 5237 T-PPG signals from 2362 unique adult patients collected between January 2020 and February 2025 at the UCSD Health System (Fig. 1).

Table 1 presents the demographics and comorbidities of our retrospective cohort used for ML analysis, broken down by ABI range. Note that PAD is defined as an ABI < 0.90. This cohort spans two medical campuses at the UCSD Health System and includes patients who were referred for a T-PPG assessment on clinical suspicion of PAD due to leg symptoms.

**Table 1:** Characteristics and comorbidities of cohort used for model development

	Total	Severe PAD	Likely PAD	Borderline PAD	Healthy	*p* Value
		ABI < 0.7	0.7 ≤ ABI < 0.9	0.9 ≤ ABI < 1.0	1.0 ≤ ABI < 1.4	
*n*	2362	466 (19.73%)	320 (13.55%)	430 (18.20%)	1146 (48.52%)	
Sex						
Male	1303	277 (21.26%)	187 (14.35%)	206 (15.81%)	633 (48.58%)	*p* = 0.017
Female	1057	189 (17.88%)	133 (12.58%)	223 (21.10%)	512 (48.44%)	
Unknown/declined to answer	2	0 (0%)	0 (0%)	1 (50%)	1 (50%)	
Mean age	69.6	73.0	70.1	69.3	68.2	*p* < 0.001
Ethnicity						
Hispanic	424	88 (20.75%)	62 (14.62%)	74 (17.45%)	200 (47.17%)	*p* = 0.294
Non-Hispanic	1871	359 (19.19%)	251 (13.42%)	340 (18.17%)	921 (49.23%)	
Unknown/declined to answer	67	19 (28.36%)	7 (10.45%)	16 (23.88%)	25 (37.31%)	
Race						
White	1523	288 (18.91%)	215 (14.12%)	276 (18.12%)	744 (48.85%)	*p* = 0.122
Black	126	34 (26.98%)	17 (13.49%)	18 (14.29%)	57 (45.24%)	
Asian	207	33 (15.94%)	17 (8.21%)	41 (19.81%)	116 (56.04%)	
Other/mixed race	459	98 (21.35%)	65 (14.16%)	87 (18.95%)	209 (45.53%)	
Unknown/declined to answer	47	13 (27.66%)	6 (12.77%)	8 (17.02%)	20 (42.55%)	
Comorbidity						
Congestive heart failure	389	94 (24.16%)	49 (12.60%)	76 (19.54%)	170 (43.70%)	*p* = 0.054
Coronary artery disease	763	195 (25.56%)	125 (16.38%)	130 (17.04%)	313 (41.02%)	*p* < 0.001
Diabetes	918	203 (22.11%)	143 (15.58%)	163 (17.76%)	409 (44.55%)	*p* = 0.003
Hypertension	1829	338 (18.48%)	262 (14.32%)	341 (18.64%)	888 (48.55%)	*p* = 0.012
Sleep apnea	631	76 (12.04%)	72 (11.41%)	117 (18.54%)	366 (58.00%)	*p* < 0.001
End-stage renal disease	113	33 (29.20%)	20 (17.70%)	11 (9.73%)	49 (43.36%)	*p* = 0.007
Smoking status						
Current smoker	249	77 (30.92%)	45 (18.07%)	41 (16.47%)	86 (34.54%)	*p* < 0.001
Former smoker	962	231 (24.01%)	171 (17.78%)	166 (17.26%)	394 (40.96%)	
Never smoker	1105	141 (12.76%)	98 (8.87%)	216 (19.55%)	650 (58.82%)	
Unknown/declined to answer	46	17 (36.96%)	6 (13.04%)	7 (15.22%)	16 (34.78%)	

Demographics, comorbidities, and smoking status descriptions for cohort of 2362 unique individuals categorized by peripheral artery disease (PAD) severity. Continuous variables are reported as means; categorical variables are presented as counts (percentages). *P*-values were calculated using one-way ANOVA for continuous variables and chi-squared for categorical variables, comparing across ankle brachial index (ABI) categories.

### Explainable PPG features correlate with ABI

As T-PPG is given per leg, and some patients had repeat assessments, our dataset of 2362 unique individuals consists of 5237 T-PPG waveforms. After featurizing the waveforms, we plotted the individual PPG feature magnitudes against the ground truth ABI value to determine if a statistically significant relationship explains the feature, or whether a given feature is not correlated with ABI. A total of 1525 PPG features were computed initially, and after feature selection (See Methods), a total of 78 features were selected for further model development. To evaluate the stability of the selected feature set across populations and measurement conditions, feature selection stability was analyzed with bootstrapping (*n* = 100) across the two datasets independently. Features were tracked across iterations to assess the consistency of selection. The 78 features used for model development demonstrated consistent representation among the top-ranked features across iterations, with mean selection frequencies of 0.707 ± 0.389 and 0.765 ± 0.310 for the La Jolla and Hillcrest datasets, respectively. Here we highlight the most noteworthy findings.

**Fig. 2:**
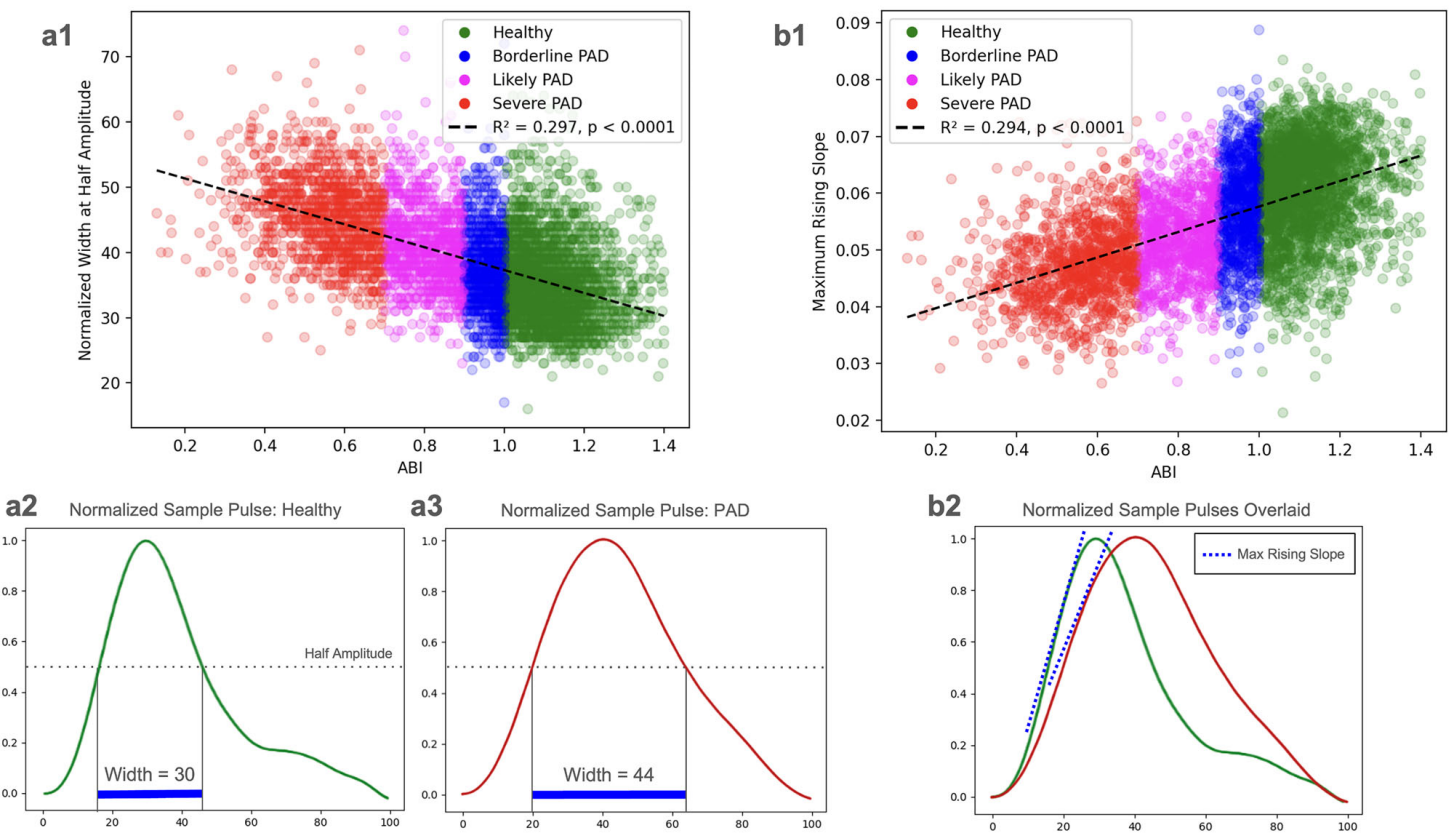
Photoplethysmography (PPG) morphology features are correlated with ankle brachial index (ABI). Data points are color-coordinated for peripheral artery disease (PAD) disease level, consistent with the thresholds in Table 1, where green is healthy, borderline PAD is blue, likely PAD is pink, and severe PAD is red. **a** PPG feature of normalized width at half amplitude is statistically correlated with ABI (**a1**) (R^2^ = 0.297, slope = −2.319, *p* < 0.0001). A sample healthy pulse (**a2**) is compared to a sample PAD pulse (**a3**), where the normalized width at half amplitude is highlighted on each in blue. **b** PPG feature of maximum rising slope is statistically correlated with ABI (**b1**) (R^2^ = 0.294, slope = 2.308, *p* < 0.0001). **b2** A sample healthy pulse (green) is compared to a sample PAD pulse (red), where the maximum rising slope is highlighted on each with the blue dotted line.

We found that the features of ‘normalized width at half amplitude’ and ‘maximum rising slope’ were two of the top-most positively/negatively correlated with ABI. In Fig. 2, we present the correlations between each of these features and ABI, where each data point represents an individual’s right or left leg and its associated PPG feature magnitude and ABI measurement. The steeper the slope in these correlation plots, the greater the correlation with ABI as the feature magnitude changes more drastically across ABI values.

Specifically, healthy PPG waveforms tend to have a narrower width at half amplitude than PAD PPG waveforms. In Fig. 2a1, we plot the correlation between the ‘normalized width at half amplitude’ feature and ABI and find a statistically significant (*p* < 0.0001) relationship with R^2^ = 0.297 and a trendline slope from the normalized feature space of −2.319. As seen, the lower the ABI value, the larger the width of the pulse. To further illustrate this, we demonstrate the differences between a healthy PPG signal (Fig. 2a2) and one indicative of PAD (Fig. 2a3). In the healthy signal, we note that the width at half amplitude is shorter than in the unhealthy signal.

Additionally, we find that healthy PPG waveforms tend to have a larger maximum rising slope than PAD PPG waveforms. In other words, healthy signals have a faster, steeper rising edge. In Fig. 2b1, we plot the correlation between the ‘maximum rising slope’ feature and ABI and find a statistically significant (*p* < 0.0001) relationship of R^2^ = 0.294 and a trendline slope from the normalized feature space of 2.308. Here, the correlation is positive, where the feature magnitude increases with increasing ABI levels. To again compare the healthy PPG signal and one indicative of PAD, we overlay these signals in Fig. 2b2 to illustrate the larger maximum rising slope on the healthier signal.

### ML model for PPG-based PAD detection

**Fig. 3:**
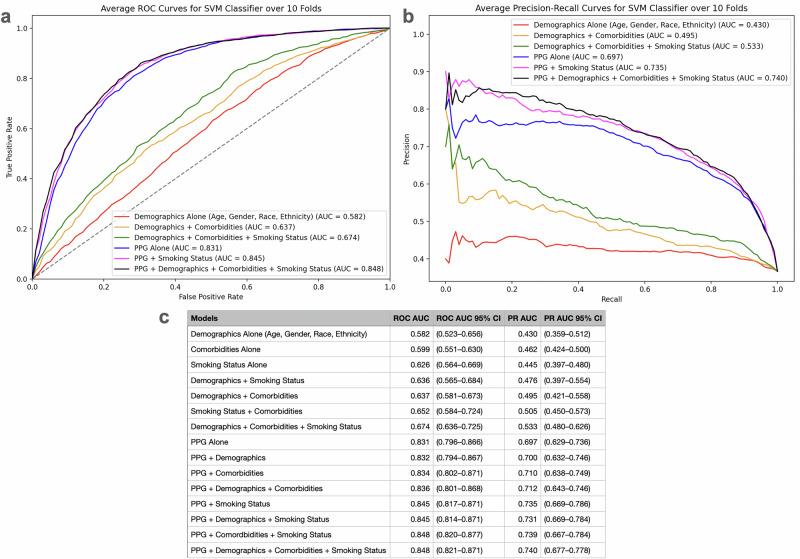
Support vector machine (SVM) performance on clinical, photoplethysmography (PPG) waveform, and combinations of clinical and waveform features. Area under the curve (AUC) performance for SVM classifier over 10 folds. Each color denotes a different combination of clinical and waveform features. Demographics alone (including age, gender, race, and ethnicity) is shown in red. Demographics and comorbidities is shown in orange. Demographics, comorbidities, and smoking status is shown in green. PPG alone is shown in blue. PPG and smoking status is shown in pink. PPG, demographics, comorbidities, and smoking status combined is shown in black. **a** All models with PPG waveform features outperform clinical-only models in the receiver operating characteristic (ROC) curve. **b** All models with PPG waveform features outperform clinical-only models in the precision-recall (PR) curve. **c** ROC-AUC and PR-AUC results with 95% confidence interval (CI) for all combinations of PPG waveform, demographics, comorbidities, and smoking status features.

We trained a support vector machine (SVM) to perform ABI-defined PAD classification using features from a T-PPG waveform. In a ten-fold cross-validation on unseen individuals, the area under the receiver operating characteristic curve (AUC) was 0.831 with a 95% confidence interval (CI) of 79.6% to 86.6% and the AUC of the precision-recall was 0.697 with a 95% CI of 62.9% to 73.6%. Figure 3 depicts the receiver operating characteristic (ROC) curve and the precision-recall (PR) curve for the SVM model trained only on PPG features (in blue, ‘PPG Alone’). For all selected 78 features for model development based on mutual information analysis, we report R^2^ and standardized slope values in Supplemental Table 1.

### Enhanced PPG-based PAD detection model

Next, we trained the SVM model with different combinations of demographics, comorbidities, smoking status, and PPG features reported in Table 1. Figure 3 presents the ROC curves for these different combinations. The SVM model using demographics alone performs the worst (AUC = 0.582, 95% CI 52.3–65.6%), followed by the model with demographics and comorbidities (AUC = 0.637, 95% CI 58.1–67.3%). Adding smoking status to this somewhat improved model performance (AUC = 0.674, 95% CI 63.6–72.5%).

In contrast, using PPG features alone created a high-performing PAD detection model (AUC = 0.831, 95% CI 79.6–86.6%). Adding smoking status to the PPG model further improved discrimination performance (AUC = 0.845, 95% CI 81.7–87.1%). Lastly, adding demographics, comorbidities, and smoking status to the PPG model similarly further improved performance (AUC = 0.848, 95% CI 82.1–87.1%). A pairwise comparison using DeLong’s test for correlated ROC curves confirms that the difference in AUC between the PPG alone model and the combined model was statistically significant (ΔAUC = 0.017, *p* < 0.001). The difference in AUC between the PPG alone model and the PPG plus smoking status model was also statistically significant (ΔAUC = 0.014, *p* < 0.001). However, the incremental AUC improvement of incorporating all clinical features compared to just adding smoking status is not statistically significant (ΔAUC = 0.003, *p* = 0.052).

The PR curves in Fig. 3 present a similar hierarchy in results with demographics alone demonstrating the lowest performance (AUC = 0.430, 95% CI 35.9–51.2%), followed by demographics and comorbidities (AUC = 0.495, 95% CI 42.1–55.8%), and then the model with demographics, comorbidities, and smoking status (AUC = 0.533, 95% CI 48.0–62.6%). Waveform features alone drastically improves PR performance (AUC = 0.697, 95% CI 62.9–73.6%). Adding smoking status to waveform features further improves performance (AUC = 0.735, 95% CI 66.9–78.6%) and adding demographics, comorbidities, and smoking status to waveform features similarly improves performance (AUC = 0.740, 95% CI 67.7–77.8%).

**Fig. 4:**
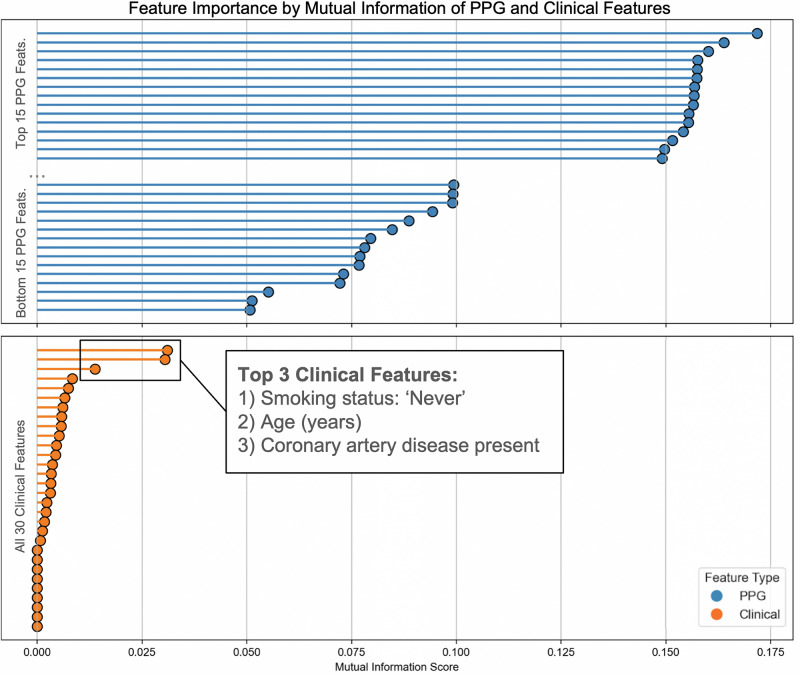
Feature importance of photoplethysmography (PPG) waveform and clinical features. Mutual information scores of PPG waveform (blue) and clinical (orange) features. All waveform features outperform all clinical features in importance. Just the top 15 and bottom 15 of waveform features are shown for clarity.

We perform mutual information analysis to inform what waveform and clinical features contribute most to the performance of the model. The larger the mutual information score, the more that feature is correlated with ABI. A mutual information score of zero implies that that feature is independent with ABI (in other words, knowing that feature does not improve the prediction of ABI). Figure 4 illustrates that all 78 waveform features have larger mutual information scores than all 30 clinical features. Of the clinical features, the most informative are a never-smoking status, their age, and whether the patient has coronary artery disease. The full list of all 108 features (78 waveform features, 30 clinical features) and their associated mutual information scores are detailed in Supplemental Table 2.

### Performance evaluation across cohorts

**Fig. 5:**
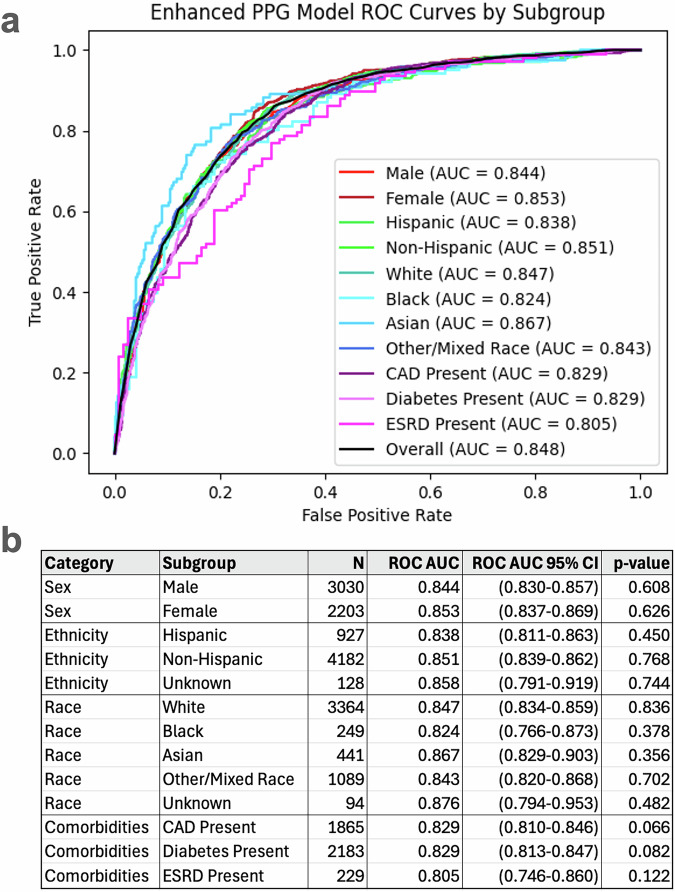
Enhanced photoplethysmography (PPG) model area under the curve (AUC) performance across cohort. **a** Receiver operating characteristic (ROC) curves for each subgroup compared to the overall model performance. Solid colored lines distinguish subgroups: Male (red), Female (crimson), Hispanic (dark green), Non-Hispanic (lime green), White (turquoise), Black (cyan), Asian (sky blue), Other/Mixed Race (dark blue), CAD Present (dark purple), Diabetes Present (pink), ESRD Present (hot pink) against Overall (black). **b** ROC-AUC with 95% confidence interval (CI) and two-sided *p*-values reported for each sex, ethnicity, race, and highlighted comorbidity subgroup. Note that subgroup performance metrics were not reported for ‘Unknown’ Sex due to insufficient sample size (*N* = 4). Abbreviations: CAD, coronary artery disease; ESRD, end-stage renal disease.

Finally, we find consistent model performance across clinical subgroups and T-PPG datasets. Figure 5 compares the ROC curves for each subgroup (sex based, ethnicity-based, race-based, and relevant comorbidity-based) against the overall model performance for the enhanced model (PPG + Demographic + Comorbidities + Smoking Status features). Figure 5b presents the 95% CI for these AUC values, the sample size of the subgroup (per T-PPG, total = 5237), and the two-sided *p*-value of the subgroup’s AUC compared to the overall AUC of the model. As seen, all *p*-values are greater than 0.05 and thus tell us that each subgroup does not have a statistically significantly different performance than the overall model performance. We also evaluated the model performance on each dataset by itself and found that compared to the combined model (ROC AUC = 0.848, 95% CI: 0.821–0.871), a Hillcrest-only dataset yields a ROC AUC of 0.856 (95% CI: 0.798–0.897) and a La Jolla-only dataset yields a ROC AUC of 0.843 (95% CI: 0.821–0.885). Further subgroup analysis on all comorbidity statuses and smoking statuses can be found in Supplementary Table 3.

## Discussion

This paper is the first to describe an explainable ML approach to detecting PAD with short-duration PPG signals. Our findings support the existence of a reproducible PPG-derived digital biomarker that captures peripheral vascular pathophysiology relevant to ABI-defined PAD. The principal findings of this study are that: (1) Specific, explainable PPG morphology is correlated with ABI/PAD; (2) our SVM model trained solely on PPG features demonstrates good discrimination of ABI-defined PAD (AUC = 0.83) consistent across diverse patient populations; (3) incorporating smoking status into the model improves performance (AUC = 0.85); (4) incorporating demographics and comorbidities into the PPG model has limited incremental value beyond waveform features and smoking status.

These findings should be interpreted in the context of ABI as the clinical reference standard used in this study and its known strengths and limitations. While abnormal ABI values are well established as prognostic markers for adverse cardiovascular and limb outcomes, ABI does not fully capture the heterogeneity of PAD^7,11–14^. Accordingly, the observed associations between short-duration, explainable toe PPG waveform morphology and ABI-defined PAD should not be interpreted as definitive clinical diagnostic validation. Because the model was trained and evaluated against ABI-defined PAD, the reported performance metrics may reflect physiologic concordance with this reference standard more than definitive detection of underlying arterial disease. The observed AUC should therefore be interpreted in the context of the known diagnostic characteristics of ABI. This distinction is particularly important in populations where ABI is known to underperform, including individuals with diabetes or chronic kidney disease. In these groups, the model’s true sensitivity for hemodynamically significant PAD may differ from reported performance. Future studies incorporating complementary physiologic reference standards, including toe-brachial index and invasive or cross-sectional imaging-based stenosis assessment, will be necessary to fully characterize diagnostic performance across the spectrum of PAD severity. Our results do demonstrate that T-PPG waveforms encode distal vascular hemodynamics that align with clinically meaningful disease states identified in routine practice. The persistence of these associations despite ABI’s known limitations supports the potential role of PPG-derived features as complementary digital biomarkers of PAD and provides a pragmatic foundation and rationale for future studies incorporating multimodal reference standards, including toe pressures, image-based assessment such as duplex ultrasound, invasive or computed tomography angiography, and longitudinal outcomes.

These findings are consistent with the underlying clinical physiology. In PAD, limb perfusion is reduced due to intraluminal plaque with restricted blood flow and less elastic arteries; the PPG signal becomes more dampened and less defined in shape. A healthy signal is characterized by a steep systolic upstroke and the presence of a dicrotic notch^23^. In individuals with stiffer arteries, the PPG morphology takes on different characteristics as the blood reflects off the vessel walls at different stages in the cardiac cycle. This understanding defines the physiologic correlations seen in our PPG features across ABI values. Specifically, we showed in Fig. 2a that healthier patients (higher ABI values) have smaller widths at half amplitude than patients with PAD. This correlates with findings that PPG signals of healthy individuals have a defined, quicker rising and falling period than patients with stiffer arteries, in which the blood flow through the vessels is more prolonged^24–27^. Similarly, Fig. 2b shows that the maximum slope of the systolic upstroke is larger for healthy patients compared to patients with PAD. This also aligns with the concept of healthy signals having a faster, defined rising edge^24,27,28^. Additionally, this concurs with previous findings that the steepness of the rising edge is correlated with PAD severity as measured by duplex ultrasound-derived pedal acceleration time (PAT)^29^. The physiological basis underlying changes in PPG waveform morphology with reduced blood flow is well established and was not the focus of novelty in this study. Rather, this existing physiologic understanding provides a robust foundation for systematic feature extraction and algorithmic modeling. The contribution of this work lies in demonstrating that known, interpretable waveform characteristics can be quantitatively extracted from short-duration toe PPG signals and integrated into an explainable machine learning framework at scale. In this respect, the novelty is methodological and translational, enabling established vascular physiology to be leveraged as a digital biomarker in a manner not previously described. While this approach provides technical explainability, translating these findings into clinically interpretable explanations (i.e., describing a waveform pattern as consistent with proximal arterial stenosis, arterial stiffness, or impaired distal perfusion) remains an important future step for clinical deployment. Bridging this gap will require prospective validation incorporating multimodal physiologic reference standards and iterative co-design with vascular specialists to ensure model outputs can be communicated in terms of pathophysiologic mechanisms and patient-specific vascular findings

Our work emphasizes the detection of ABI-defined PAD via PPG signals using an explainable ML framework grounded in physiologically interpretable waveform features. Relating our informative features to the fundamental processes that define PAD allows us to develop ML models that are grounded in physiological meaning, rather than a black-box approach. Further, we used an SVM rather than a neural net for model development as it further emphasizes this explainable approach. We are using known, explainable features to generate our results.

We find that all waveform features outperform clinical features in importance to our SVM model. We believe this is due to a few reasons. Firstly, we already selected only top-performing waveform features (78 out of 1525) for model development. If we incorporated all waveform features, the clinical features would likely outperform the waveform features that we filtered out. Secondly, we had a population with a high pre-test probability of PAD. Our cohort is comprised of patients already presenting for a T-PPG assessment, and thus, this population is already an at-risk population for PAD. Therefore, the fact that our demographics and comorbidities features do not vastly improve the model implies that comorbidities do not adequately discriminate risk in an already high-risk population. If the study cohort were more representative of a general population (and not an at-risk population), then it is possible that demographics and comorbidities could improve model performance.

It is also noteworthy that smoking status does improve our waveform-based approach. Physiologically, our findings align with the fact that smoking is a major independent risk factor for PAD, with smokers having approximately double the risk compared to non-smokers. Additionally, smoking is known to accelerate arterial stiffness in a dose-dependent manner^30–33^. Further work should be done to determine whether the importance of smoking is universal or unique to our dataset.

As expected, participants with lower ABI values tended to be older and had a higher prevalence of traditional cardiovascular comorbidities such as coronary artery disease (CAD), diabetes, and end-stage renal disease (ESRD). These descriptive trends mirror the established epidemiology of PAD, supporting that the cohort captures the full clinical spectrum of disease severity. Although *p*-values are presented for completeness, these comparisons were intended to describe rather than test hypotheses about group differences. The observed gradients across ABI strata provide clinical context for interpreting subsequent analyses and reinforce the biological plausibility of the associations identified by our digital biomarker model.

Our work builds upon prior studies that have largely relied on deep neural networks (DNNs) or small, descriptive feature sets for PPG analysis^20,27,28,34^. These earlier efforts were limited by small sample sizes, required long waveform acquisition times, and typically assessed only a few features, constraining both interpretability and scalability. In contrast, we analyzed 1525 waveform features using the largest PPG dataset with concurrent ABI measurements to date (5237 waveforms from 2362 distinct patients). Importantly, our framework requires only 4 s of PPG signal to extract informative features, distinguishing it from prior approaches that depend on substantially longer recordings or continuous monitoring. Together, these advances enable broader application of a scalable source of vascular physiologic information to potentially identify ABI-defined PAD and support cardiovascular risk stratification^13–15^.

As PPG is an optical-based measurement, it is important to note that we find no statistically significant differences in performance across race or ethnic groups. Although comorbidities such were more prevalent among participants with lower ABI values, the model demonstrated consistent performance across key clinical subgroups, including patients with ESRD and diabetes, both of which make diagnosing PAD particularly challenging using traditional methods^12,21,32,35–38^. The algorithm maintained high accuracy across these high-risk groups, highlighting its potential utility in these populations. To our knowledge, this is the first study to evaluate and report the performance of a PPG-based ABI-defined PAD detection model specifically in patients with ESRD and diabetes. While we were not able to develop a PPG-based model in high-risk groups that had non-compressible arteries due to our exclusion criteria, we were able to identify PAD status in patients with high-risk disease processes such as diabetes and ESRD who did have compressible tibial vessels. Additional research should validate the performance of these groups when non-compressible arteries are included by using other measures such as toe pressure and/or arterial waveform data. Moreover, this is the first study to include a substantial proportion of Hispanic individuals (424 individuals comprising 17.95% of the study population) and Black individuals (126 individuals comprising 5.33% of the study population), addressing long-standing gaps in digital biomarker research and enhancing the generalizability and equity of model performance across diverse patient populations. However, it is still important to note that despite the larger number of Black individuals compared to other studies, the overall cohort size remains limited and may restrict broader generalizability across populations. Additionally, we find that our results are consistent across databases, which suggests that our algorithm is robust to varying clinical T-PPG assessment equipment, location, and personnel. While both databases fall within the UCSD Health System, the machines used for collection come from different manufacturers and are staffed by different technicians. Therefore, while future work should be done to validate results consistency across institutions, we demonstrate early evidence that the results are not necessarily dependent on specific environmental factors.

Importantly, the present findings are not intended to represent the final stage of validation, but rather to establish the physiologic and methodological foundation required for prospective evaluation. Based on the robustness of short-duration signal acquisition, feature interpretability, and performance consistency observed in this study, this framework is now the foundation for prospective studies designed to assess performance across clinical workflows and complementary reference standards. These studies will enable evaluation under real-world conditions, incorporation of additional physiologic comparators, and assessment of downstream clinical utility, thereby advancing this digital biomarker from retrospective characterization toward translational application.

We must acknowledge some final limitations of our work. First, our study cohort was limited to a single health system, and future prospective multi-center validation is needed. Second, the population comprised of patients already presenting for a clinical PAD assessment, and thus the cohort has higher rates of comorbidities and PAD prevalence than the general population. This limits the generalizability of our study and future work is necessary to prospectively evaluate the findings in the general population. Third, PPG signals were acquired under controlled vascular laboratory conditions; performance on PPG waveforms in unsupervised or consumer-grade environments is unknown. Fourth, although our study included patients from diverse racial and ethnic backgrounds and demonstrated comparable model performance across these groups, we did not formally assess skin tone or its potential impact on PPG signal quality and model accuracy. Given that melanin content can affect PPG optical signal absorption, future studies should incorporate standardized measures of skin tone to evaluate and ensure equitable performance of PPG-based models across the full spectrum of pigmentation^39,40^. Additionally, PAD was determined in this study as ABI < 0.90. More realistically, PAD is diagnosed as a constellation of objective findings (ABI) and patient signs and symptoms. Future work can use multimodal data (e.g., patient symptoms and/or invasive angiography) to determine the performance of our algorithm on blood flow obstruction and symptomology. Our study did not include patients with non-compressible arteries, as it is impossible to determine a patient’s PAD diagnosis based on ABI if their arteries are non-compressible. Those with non-compressible ABIs are an important patient population to address, as they have a high risk of cardiovascular events. Therefore, future work should be performed to determine the ability of a PPG-based model in assessing for PAD in non-compressible patient populations. Such studies could provide interpretable metrics of vascular health, extending utility to populations in which arterial non-compressibility limits ABI accuracy. Finally, although PPG waveforms were routinely collected as part of vascular laboratory assessments, contemporaneous toe pressure measurements were not consistently performed or recorded in structured form across both clinical sites and equipment vendors. As a result, toe pressure values could not be used as a uniform comparator in this retrospective analysis. Future prospective studies incorporating standardized toe pressure acquisition alongside PPG waveform analysis will be important to further define the diagnostic and prognostic utility of PPG-derived digital biomarkers. Prospective deployment studies are underway to evaluate performance across clinical settings and reference standards, which will be necessary to determine real-world applicability.

Lastly, false positives in PAD screening may lead to anxiety and patient burden on healthcare resources. Future work is necessary to study the realistic deployment of the screening model and how access to this tool improves widespread PAD screening while examining costs to the health system. Special care should be taken to study underserved populations’ retention and whether PPG-based PAD screening is an appropriate and reasonable tool for addressing the disproportionate effects of PAD across communities.

## Methods

### Study population and data collection

This retrospective cohort utilized de-identified clinical and waveform data from two campuses of the UCSD Health System. Adult patients aged 18 years or older who underwent both ABI testing and T-PPG assessment as part of their PAD evaluation between January 2020 and February 2025 were eligible for inclusion. Patients were excluded if they had incomplete waveform data, missing ABI results, or insufficient clinical metadata. This work and all methods included in this study were approved by the institutional review board (IRB) of the University of California San Diego (IRB Approval #809658) and were granted a waiver of informed consent due to the use of de-identified data and minimal risk to participants. All research procedures were conducted in accordance with institutional and national research committee ethical standards and with the principles outlined in the Declaration of Helsinki.

Raw T-PPG waveform signals were extracted from the vascular laboratory database at each site. All waveforms were obtained using standard non-invasive vascular testing protocols. Prior to analysis, waveform data underwent preprocessing, including noise removal and standardization of amplitude and time scales to ensure consistency across acquisition systems. The ABI was used as the reference standard for PAD diagnosis, with PAD defined as an ABI < 0.90. ABI values between 0.90 and 1.40 were considered normal. Values above 1.40, suggestive of non-compressible arteries, were excluded. ABI studies were also excluded if they were textually labeled non-compressible. Demographic data including age, sex, race, and ethnicity, as well as clinical comorbidities such as diabetes mellitus, heart failure, and end-stage renal disease (ESRD), were extracted from linked electronic health records (EHRs). Comorbidities were defined based on International Classification of Diseases (ICD-9/10) codes recorded within 12 months of the vascular evaluation.

The ABI was calculated from the raw blood pressure measurements provided in the T-PPG assessment. We used sensitive criteria for ABI, and as such, the lower blood pressure between the posterior tibial and dorsalis pedis was used in combination with the maximum left or right brachial blood pressure^30^. ABI was calculated per leg. The TBI was calculated as the ratio of the blood pressure between the toe (digit 1) and the maximum brachial blood pressure.

### PPG waveform preprocessing

All T-PPG waveforms were pre-processed consistently. First, the signals were resampled to 75 Hz, to ensure consistency across the dataset. Next, a band-pass filter between 0.5 and 8 Hz was applied. This frequency range was selected to filter out artifact noise from breathing or other movements, as well as high-frequency noise from sensing equipment. Finally, waveforms with their main frequency components outside the physiological expected range for heart rate were classified as noise and discarded.

### PPG feature extraction

All timeseries waveforms were four seconds in duration. To generate features for each waveform, one representative pulse per four-second signal was generated. This pulse was then normalized in amplitude (starting amplitude at zero and peak at one) and relative time (interpolated for 100 points). Features of amplitude and time were calculated prior to normalization to preserve the raw morphology of the pulse. All other features were calculated after normalization. Features in seven different categories were constructed: rise features, fall features, derivative features, width features, symmetry features, miscellaneous features (e.g., area under the curve), and TSFEL features (TSFEL features were generated automatically using Python’s Time Series Feature Extraction Library). Supplemental Fig. 2 depicts example features for each six categories of computed features (not including TSFEL). 1,525 total features were generated for each waveform.

### Feature selection

To identify PPG features most associated with PAD, we performed mutual information analysis between each generated candidate feature and ABI. Mutual information scores were computed using the mutual_info_classif function from the sklearn feature selection library. To improve robustness and reduce the likelihood of dataset-specific feature selection, mutual information analysis was performed independently across each dataset. Within each feature category, features ranking in the top 25% by mutual information were retained for that dataset. Features were then compared across datasets, and only those consistently ranking within the top quartile across datasets were preserved. To further promote robustness to differences in clinical labeling, feature informativeness was also evaluated for consistency across analytic conditions, and features demonstrating stable rankings were retained. Only features meeting the top-quartile threshold across datasets and exhibiting consistent informativeness under available definitions were retained, resulting in a final set of 78 features used for model development. To assess the stability of the selected feature set, bootstrap resampling (*n* = 100) was performed independently for each dataset within the ABI-defined cohort. In each iteration, mutual information was recomputed, and features were re-ranked using the same category-wise top quartile selection framework. The frequency with which each feature was retained across bootstrap iterations was used as a measure of feature selection stability.

### SVM model development

We built a linear SVM with 78 features (as described previously) of PPG waveform, and truth values given as that waveform’s ABI. To enhance the model with clinical data, categorical variables are one-hot encoded and converted to integers to serve as additional model features. All model features are consistently scaled, using the standard scaler, to avoid any bias from a feature’s magnitude. Sklearn’s StratifiedGroupKFold was used to ensure that individuals stay within their own fold and thus validation is always performed on unseen individuals from the training cohort. The SVM was built with a linear kernel, balanced class weights, and regularization parameter C of 0.05. To maximize analysis, 10-fold cross validation was performed instead of using a hold-out validation set. Reported hit rates, receiver operator curve results, and precision-recall results are averaged across the 10 results. 95% confidence intervals for ROC AUC and PR AUC were computed from the distribution of fold-level scores, defined as the 2.5th and 97.5th percentiles of the cross-validation results. Differences in AUC between models were assessed using DeLong’s test for correlated ROC curves.

### Mutual information feature importance

We calculate mutual information as a metric for how correlated each feature is with the ground truth ABI value. We use the standard scaler on all features prior to mutual information calculation to ensure all features are properly weighted. Additionally, mutual information is calculated when all features are incorporated into the feature space so they all scale in the same ratio. The raw mutual information number itself is less informative than the ordering and relative size of the score compared to other features.

### Statistical analysis

Baseline demographic and clinical characteristics in Table 1 were compared across ankle–brachial index (ABI) categories, defined as severe PAD (ABI < 0.7), likely PAD (0.7 ≤ ABI < 0.9), borderline PAD (0.9 ≤ ABI < 1.0), and healthy (1.0 ≤ ABI < 1.4). Continuous variables were expressed as means and compared using one-way analysis of variance (ANOVA). Categorical variables were summarized as counts and percentages and compared using chi-squared, as appropriate. A two-sided *p* < 0.05 was considered statistically significant.

### Subgroup analysis

Model performance was further evaluated across subgroups defined by sex, race, ethnicity, comorbidity statuses, and smoking status to assess clinical generalizability, particularly in underserved populations. For each subgroup, the SVM model trained with all PPG and clinical features was used to generate predicted probabilities and observed labels. Subgroup-specific ROC curves were computed, and the AUC was calculated for each subgroup. To estimate 95% confidence intervals for the AUC, we used bootstrap resampling where 1000 samples were drawn with replacement within each subgroup to generate a distribution of AUCs and the 2.5th and 97.5th percentiles of this distribution were taken as the confidence bounds. A two-sided *p*-value was also computed to compare each subgroup’s AUC distribution against the bootstrapped distribution of the overall cohort, providing a measure of whether the subgroup performance differed significantly from the overall model performance (if *p* < 0.05, then there is a statistically significant difference).

## Supplementary information

Supplementary information

## Data Availability

The data used in this study were collected as part of routine clinical care at the UCSD Health System and are considered proprietary to UCSD. Due to institutional policies and patient privacy regulations, the dataset is not publicly available. However, access to de-identified data may be granted for non-commercial academic research purposes through a data use agreement and approval by the UC San Diego Office of Compliance and the Office of Innovation and Commercialization. Interested investigators may contact the corresponding author to initiate the data sharing process.

## Code availability

The underlying code for this study is not publicly available for proprietary reasons. Following resolution of IP protection and commercialization strategy, we intend to release portions of the code as appropriate under a suitable license.
