# Supplementary material for: Development and validation of a digital biomarker for peripheral artery disease
Source: NPJ Digit Med. 2026 May 12;9:563. doi: 10.1038/s41746-026-02655-w (PMC13389492; doi:10.1038/s41746-026-02655-w)
Supplement: Supplementary file 1 — Supplementary information [file 41746_2026_2655_MOESM1_ESM.pdf]

Supplementary Materials

Supplemental Figure 1: Characteristics of Excluded Signals.

Excluded signals do not bias the dataset toward certain patient groups or PAD severity levels as the distribution of races, ethnicities, and ABI levels are consistent between the full dataset (blue) and the excluded signals (orange). In other words, the excluded signals are representative of the full dataset across disease level and racial/ethnicity demographics.

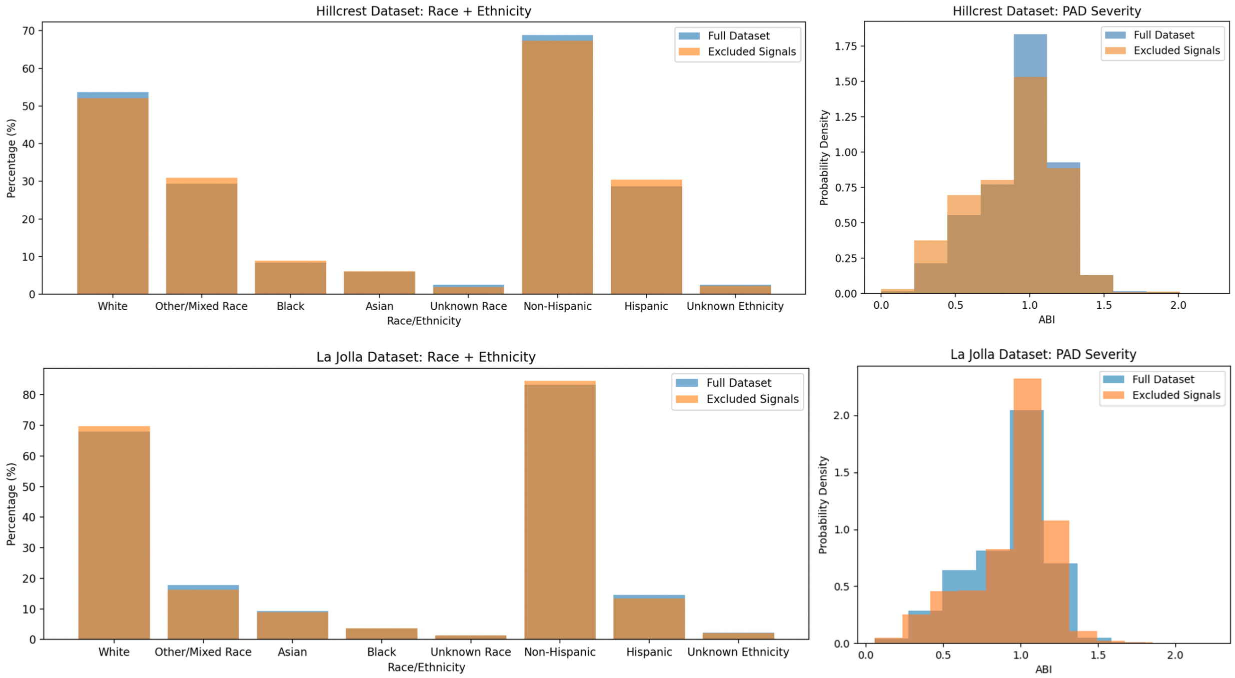

Supplemental Figure 2: Example PPG Morphology Features for Six Computed Categories.

Six of the same normalized example pulse are shown. On each, an example feature(s) from the given category are visualized. Top left: rise time features. Top middle: fall time features. Top right: width features. Bottom left: symmetry features. Bottom middle: derivative features. Bottom right: miscellaneous features.

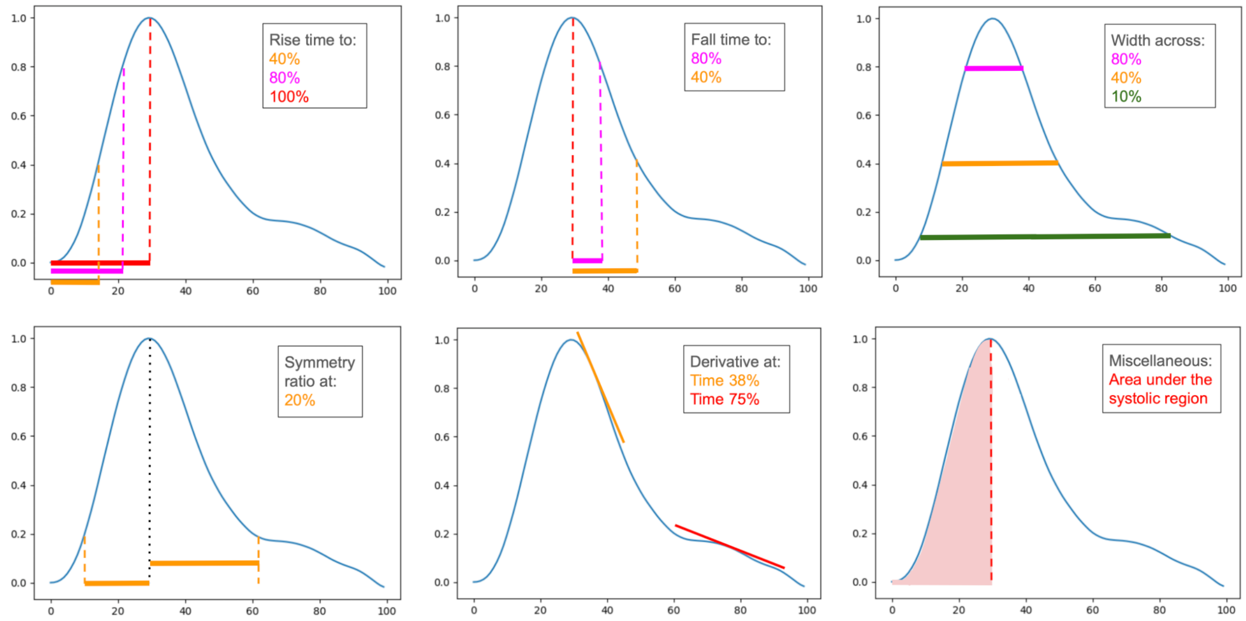

Supplemental Table 1: Feature Correlations for 78 Photoplethysmography Features used in Model Development.

Extensive list of photoplethysmography (PPG) morphology features used in model development and their correlation with ABI. For each feature, the R^2 value, p-value, and the slope in the normalized feature space is reported for the given feature's relationship with ABI.

Feature Name	R Squared	Standardized Slope	p-value
Wavelet_energy_3.57Hz	0.3306	2.4464	p < 0.0001
Wavelet_standard_deviation_3.57Hz	0.3301	2.4444	p < 0.0001
fall_to_50_pct	0.3068	-2.3564	p < 0.0001
Wavelet_standard_deviation_5.0Hz	0.3054	2.3512	p < 0.0001
fall_to_60_pct	0.3048	-2.349	p < 0.0001
Wavelet_energy_5.0Hz	0.3048	2.349	p < 0.0001
width_across_60_pct	0.2986	-2.3249	p < 0.0001
width_across_50_pct	0.297	-2.3186	p < 0.0001
Skewness	0.2951	2.3113	p < 0.0001
fall_to_40_pct	0.2946	-2.3091	p < 0.0001
rise_slope_feats_maximum	0.2943	2.3079	p < 0.0001
fall_to_70_pct	0.2937	-2.3057	p < 0.0001
width_across_70_pct	0.2878	-2.2823	p < 0.0001
Spectrogram_mean_coefficient_11.29Hz	0.2877	-2.2821	p < 0.0001
Average_power	0.2867	-2.2781	p < 0.0001
Absolute_energy	0.2867	-2.2781	p < 0.0001
30_to_100_risetime	0.2861	-2.2756	p < 0.0001
Root_mean_square	0.2849	-2.2709	p < 0.0001
40_to_100_risetime	0.282	-2.2593	p < 0.0001
Wavelet_variance_5.0Hz	0.2808	2.2547	p < 0.0001
width_across_40_pct	0.2799	-2.2509	p < 0.0001
fall_to_80_pct	0.2784	-2.245	p < 0.0001
20_to_100_risetime	0.2739	-2.2267	p < 0.0001
50_to_100_risetime	0.2724	-2.2203	p < 0.0001
Spectrogram_mean_coefficient_12.9Hz	0.2684	-2.2042	p < 0.0001

30_to_90_risetime	0.2677	-2.2014	$p < 0.0001$
Spectral_entropy	0.266	2.1942	$p < 0.0001$
40_to_90_risetime	0.265	-2.19	$p < 0.0001$
fall_to_30_pct	0.2648	-2.1895	$p < 0.0001$
10_to_100_risetime	0.2645	-2.188	$p < 0.0001$
fall_slope_feats_median	0.2638	-2.1851	$p < 0.0001$
Median	0.2609	-2.1733	$p < 0.0001$
width_across_80_pct	0.2606	-2.1721	$p < 0.0001$
Area_under_the_curve	0.2605	-2.1714	$p < 0.0001$
Spectral_decrease	0.258	2.1612	$p < 0.0001$
Centroid	0.2578	-2.16	$p < 0.0001$
filt_deriv_at_40_pct	0.2554	-2.1502	$p < 0.0001$
Median_absolute_diff	0.2518	-2.1349	$p < 0.0001$
deriv_at_40_pct	0.2504	-2.129	$p < 0.0001$
Signal_distance	0.2487	2.1219	$p < 0.0001$
width_across_30_pct	0.2475	-2.1167	$p < 0.0001$
30_to_80_risetime	0.2473	-2.1159	$p < 0.0001$
20_to_90_risetime	0.2459	-2.1096	$p < 0.0001$
misc_feats_AUC	0.2433	-2.0984	$p < 0.0001$
Mean	0.2426	-2.0954	$p < 0.0001$
ECDF_Percentile_1	0.2418	-2.0922	$p < 0.0001$
60_to_90_risetime	0.2389	-2.0794	$p < 0.0001$
Median_absolute_deviation	0.2336	-2.0562	$p < 0.0001$
10_to_90_risetime	0.233	-2.0536	$p < 0.0001$
filt_deriv_at_70_pct	0.2304	2.0421	$p < 0.0001$
rise_to_peak	0.2276	-2.0295	$p < 0.0001$
fall_peak_to_60_pct	0.2235	-2.0113	$p < 0.0001$
rise_slope_feats_median	0.223	2.0091	$p < 0.0001$
20_to_80_risetime	0.2198	-1.9945	$p < 0.0001$
fall_peak_to_70_pct	0.214	-1.968	$p < 0.0001$
Spectrogram_mean_coefficient_14.52Hz	0.2109	-1.9539	$p < 0.0001$

Spectrogram_mean_coefficient_4.84Hz	0.2071	1.9361	p < 0.0001
10_to_80_risetime	0.2069	-1.9354	p < 0.0001
deriv_at_70_pct	0.2025	1.9144	p < 0.0001
rel_misc_feats_med_rising_to_falling_slope	0.2025	1.9145	p < 0.0001
Kurtosis	0.2015	1.9096	p < 0.0001
Spectral_distance	0.1912	1.8604	p < 0.0001
20_to_70_risetime	0.1908	-1.8584	p < 0.0001
rise_to_90_pct	0.1887	-1.8481	p < 0.0001
Slope	0.1814	-1.812	p < 0.0001
10_to_70_risetime	0.1798	-1.8038	p < 0.0001
Spectrogram_mean_coefficient_6.45Hz	0.1782	1.7959	p < 0.0001
Spectrogram_mean_coefficient_0.0Hz	0.1549	1.6744	p < 0.0001
10_to_60_risetime	0.1516	-1.6563	p < 0.0001
fall_peak_to_90_pct	0.1483	-1.6383	p < 0.0001
80_to_60_falltime	0.1432	-1.6101	p < 0.0001
Spectrogram_mean_coefficient_3.23Hz	0.1394	1.5886	p < 0.0001
Spectrogram_mean_coefficient_8.06Hz	0.132	1.5456	p < 0.0001
Median_frequency	0.1079	1.3977	p < 0.0001
30_to_20_rel_width	0.0976	-1.3295	p < 0.0001
Interquartile_range	0.0924	-1.2934	p < 0.0001
LPCC_0	0.0109	-0.4451	p < 0.0001
symm_at_50_pct	0.0019	0.1863	p = 0.0015

Supplemental Table 2: Model Features and their Mutual Information Scores.

Ranked by mutual information (MI) importance, each feature used in the enhanced model are reported by their feature type along with their associated MI Score. Abbreviations: PPG, photoplethysmography.

Feature Name	Feature Type	MI Score
rel_misc_feats_med_rising_to_falling_slope	PPG	0.1717
Wavelet_standard_deviation_3.57Hz	PPG	0.1638
Wavelet_energy_5.0Hz	PPG	0.1601
30_to_90_risetime	PPG	0.1575
Wavelet_energy_3.57Hz	PPG	0.1574
Wavelet_standard_deviation_5.0Hz	PPG	0.1573
Wavelet_variance_5.0Hz	PPG	0.1568
fall_slope_feats_median	PPG	0.1567
fall_to_60_pct	PPG	0.1565
fall_to_40_pct	PPG	0.1554
20_to_90_risetime	PPG	0.1553
30_to_100_risetime	PPG	0.1541
20_to_100_risetime	PPG	0.1515
fall_to_50_pct	PPG	0.1496
20_to_80_risetime	PPG	0.1491
rise_slope_feats_maximum	PPG	0.149
10_to_90_risetime	PPG	0.1487
width_across_60_pct	PPG	0.1474
30_to_80_risetime	PPG	0.1472
10_to_100_risetime	PPG	0.1469
rise_slope_feats_median	PPG	0.1457
40_to_90_risetime	PPG	0.1454
fall_to_70_pct	PPG	0.144
fall_to_80_pct	PPG	0.1437
Spectral_entropy	PPG	0.1434
filt_deriv_at_70_pct	PPG	0.143
filt_deriv_at_40_pct	PPG	0.1422
40_to_100_risetime	PPG	0.1421
Skewness	PPG	0.141
Centroid	PPG	0.1397
width_across_50_pct	PPG	0.1386
Signal_distance	PPG	0.1384
Spectrogram_mean_coefficient_11.29Hz	PPG	0.1383

50_to_100_risetime	PPG	0.1378
Root_mean_square	PPG	0.1376
60_to_90_risetime	PPG	0.1369
Average_power	PPG	0.1366
Absolute_energy	PPG	0.1365
10_to_80_risetime	PPG	0.1362
Median_absolute_diff	PPG	0.1358
20_to_70_risetime	PPG	0.1353
width_across_40_pct	PPG	0.1353
deriv_at_40_pct	PPG	0.1303
Kurtosis	PPG	0.1292
10_to_70_risetime	PPG	0.1284
width_across_70_pct	PPG	0.1283
rise_to_peak	PPG	0.1272
Area_under_the_curve	PPG	0.1265
fall_to_30_pct	PPG	0.1253
Spectrogram_mean_coefficient_12.9Hz	PPG	0.1246
deriv_at_70_pct	PPG	0.1234
Median_absolute_deviation	PPG	0.123
Median	PPG	0.1229
Spectral_decrease	PPG	0.1223
width_across_80_pct	PPG	0.1207
10_to_60_risetime	PPG	0.1199
width_across_30_pct	PPG	0.1167
ECDF_Percentile_1	PPG	0.1151
rise_to_90_pct	PPG	0.1129
Mean	PPG	0.1126
Spectrogram_mean_coefficient_4.84Hz	PPG	0.1121
misc_feats_AUC	PPG	0.1084
Slope	PPG	0.1012
fall_peak_to_70_pct	PPG	0.0993
fall_peak_to_60_pct	PPG	0.0991
Spectrogram_mean_coefficient_6.45Hz	PPG	0.099
Spectrogram_mean_coefficient_14.52Hz	PPG	0.0943
symm_at_50_pct	PPG	0.0887
80_to_60_falltime	PPG	0.0847
30_to_20_rel_width	PPG	0.0795
Spectrogram_mean_coefficient_0.0Hz	PPG	0.0781
Spectral_distance	PPG	0.0769

Spectrogram_mean_coefficient_8.06Hz	PPG	0.0767
fall_peak_to_90_pct	PPG	0.073
Spectrogram_mean_coefficient_3.23Hz	PPG	0.0722
Interquartile_range	PPG	0.0551
LPCC_0	PPG	0.0512
Median_frequency	PPG	0.0507
Smoking status_Never	Clinical	0.031
Age at measurement (years)	Clinical	0.0304
Coronary artery disease present	Clinical	0.0138
Smoking status_Every Day	Clinical	0.0083
Gender_Unknown	Clinical	0.0074
Hypertension present	Clinical	0.0065
Gender_Female	Clinical	0.0061
Diabetes present	Clinical	0.0058
Smoking status_Heavy Smoker	Clinical	0.0057
End-stage renal disease present	Clinical	0.0052
Ethnicity_Other Hispanic, Latino(a) or Spanish origin	Clinical	0.0045
Smoking status_Former	Clinical	0.0043
Ethnicity_Unreported (Patient refused or chose not to disclose)	Clinical	0.0036
Race 1_American Indian or Alaska Native	Clinical	0.0033
Smoking status_Never Assessed	Clinical	0.0032
Smoking status_Smoker, Current Status Unknown	Clinical	0.0031
Smoking status_Some Days	Clinical	0.0022
Gender_Male	Clinical	0.0021
Race 1_White	Clinical	0.0017
Race 1_Asian	Clinical	0.0012
Sleep apnea present	Clinical	0.0007
Congestive heart failure present	Clinical	0
Ethnicity_Not Hispanic, Latino(a), or Spanish origin	Clinical	0
Race 1_Black or African American	Clinical	0
Race 1_Native Hawaiian or Other Pacific Islander	Clinical	0
Race 1_Other Race or Mixed Race	Clinical	0
Race 1_Unknown (Patient cannot or refuses to declare race)	Clinical	0
Smoking status_Light Smoker	Clinical	0
Smoking status_Passive Smoke Exposure - Never Smoker	Clinical	0
Smoking status_Unknown	Clinical	0

Supplemental Table 3: Detailed Subgroup Analysis across All Demographic, Comorbidity, and Smoking Status Categories.

Receiver operating characteristic (ROC) area under the curve (AUC) performance listed for each subgroup compared to the overall model performance. Also, the 95% confidence interval (CI) of the ROC AUC and two-sided p-values are reported for each sex, ethnicity, race, comorbidity, and smoking status subgroup. Note that subgroup performance metrics were not reported for 'Unknown' Sex due to insufficient sample size. Abbreviations: CHF, congestive heart failure; CAD, coronary artery disease; ESRD, end-stage renal disease.

Category	Subgroup	N	ROC AUC	ROC AUC 95% CI	p-value
Sex	Male	3030	0.844	(0.830-0.857)	0.608
Sex	Female	2203	0.853	(0.837-0.869)	0.626
Sex	Unknown	4	NA	NA	NA
Ethnicity	Hispanic	927	0.838	(0.811-0.863)	0.450
Ethnicity	Non-Hispanic	4182	0.851	(0.839-0.862)	0.768
Ethnicity	Unknown	128	0.858	(0.791-0.919)	0.744
Race	White	3364	0.847	(0.834-0.859)	0.836
Race	Black	249	0.824	(0.766-0.873)	0.378
Race	Asian	441	0.867	(0.829-0.903)	0.356
Race	Other/Mixed Race	1089	0.843	(0.820-0.868)	0.702
Race	Unknown	94	0.876	(0.794-0.953)	0.482
Comorbidities	CHF Present	902	0.822	(0.794-0.849)	0.070
Comorbidities	CAD Present	1865	0.829	(0.810-0.846)	0.066
Comorbidities	Diabetes Present	2183	0.829	(0.813-0.847)	0.082
Comorbidities	Hypertension Present	4071	0.845	(0.832-0.857)	0.656
Comorbidities	Sleep Apnea Present	1351	0.841	(0.818-0.863)	0.622
Comorbidities	ESRD Present	229	0.805	(0.746-0.860)	0.122
Smoking Status	Current Smoker	599	0.828	(0.796-0.858)	0.244
Smoking Status	Former Smoker	2356	0.829	(0.813-0.845)	0.052
Smoking Status	Never Smoker	2169	0.835	(0.816-0.855)	0.262
Smoking Status	Unknown	113	0.812	(0.722-0.888)	0.410
